# *Eucalyptus cinerea* phytoactive compounds alleviate pulmonary fibrosis by targeting oxidative stress, inflammation, and immune pathways in male rats

**DOI:** 10.14814/phy2.70934

**Published:** 2026-06-25

**Authors:** Saber Jedidi, Nourhène Dhawefi, Anouar Abidi, Houcem Sammari, Ala Ayari, Hichem Sebai

**Affiliations:** ^1^ Laboratory of Functional Physiology and Valorization of Bio‐Resources, Higher Institute of Biotechnology of Beja University of Jendouba Béja Tunisia; ^2^ University of Jendouba, National Institute of Technologies and Sciences of Kef (INTeK) Kef Tunisia

**Keywords:** anti‐inflammatory, *eucalyptus cinerea*, functional foods, pulmonary fibrosis, ROS

## Abstract

*Eucalyptus cinerea* is known for anti‐inflammatory/antioxidant properties in Tunisian traditional medicine, but research on its seeds for the treatment of pulmonary fibrosis (PF) is insufficient. The study investigated the phytochemical properties and the curative potential of *Eucalyptus cinerea* seeds aqueous extract (ECAE) against bleomycin (BLEO)‐induced sub‐acute experimental PF as well as the mechanisms implicated in such protection. Firstly, the phytochemical analysis of ECAE revealed the presence of 7 phenolic compounds, including 3 phenolic acids, 3 flavonoids, and 1 tannin. Results of the in vivo assay showed that ECAE treatment significantly reduced BLEO‐induced lung lesions and improved histopathological alterations. ECAE attenuated BLEO‐induced plasma and pulmonary lipid peroxidation and the depletion of both enzymatic and non‐enzymatic antioxidants. More importantly, intratracheal instillation of BLEO increased hydrogen peroxide and calcium levels, while the ECAE treatment reversed all intracellular mediator perturbations. Also, ECAE exhibited anti‐inflammatory and immunomodulatory properties. These results suggest that *Eucalyptus cinerea* seeds contain anti‐inflammatory compounds that could help fight PF, making them a potential ingredient for functional foods.

## Graphical abstract

*Eucalyptus cinerea* fruits aqueous extract improves fibrotic parameters and attenuates oxidative stress and pulmonary inflammation in rats treated with bleomycin.

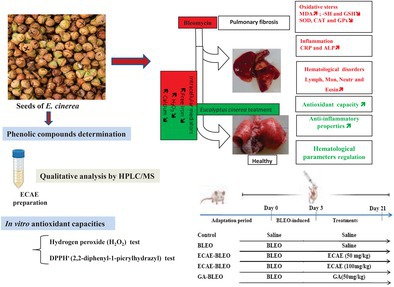

## 1 INTRODUCTION

“Green medicine”, referring to herbal/traditional remedies, is a vital, accessible, and often primary healthcare source in developing countries due to its cultural acceptance, affordability, and availability. Indeed, different parts of plants, with high levels of bioactive molecules such as secondary metabolites, fatty acids, and mineral elements, play an important role in a healthy diet, with the potential to prevent various diseases (Mitra et al., 2022). These phytomolecules offer beneficial effects by modulating key physiological pathways, thereby preventing and managing certain metabolic disorders (Ghosh et al., 2021).

Among these plants, the species *Eucalyptus cinerea* F. Muell. ex Benth (Myrtaceae family) is widely cultivated in certain regions of Tunisia thanks to its multiple beneficial properties. The main products obtained from *Eucalyptus* are oils, gum, cellulose, and wood. The bioactive compounds in this plant, such as polyphenols and essential oils, possess significant expectorant, antispasmodic, and antiviral properties, making them valuable pharmaceutical ingredients for treating respiratory ailments (Silva et al., 2011). Furthermore, it has been documented that bioactive substances isolated from the different parts of the plant exhibit multiple biological activities, including antioxidant and anti‐inflammatory properties, and that they relieve muscle and joint pain (Chen et al., 2014; Jedidi et al., 2024; Salehi et al., 2019).

Pulmonary fibrosis (PF) is a progressive lung disease characterized by destruction of the parenchyma structure. It has been shown that the alterations in lung tissue, particularly the alveolar epithelium, exerted a reduction of ventilation, leading to mucus accumulation, fibroblast‐driven tissue scarring, and progressive destruction of the tissue architecture of the alveoli and pneumocytes. These complications generally lead to the patient's death. PF is more common in men than women, with an average age at diagnosis of 65. It is currently estimated that there are approximately three million people suffering from this disease worldwide, and this figure is expected to double by 2030 (Hutchinson et al., 2015).

Although the exact origin of PF remains unknown, environmental factors, such as smoking, viral infection, chronic inhalation of wood, metal or silica particles, as well as the use of certain medications, and genetic factors, including genes associated with telomere regulation and surfactant protein production, are associated with an increased risk of developing PF (Ranzieri et al., 2019).

Current treatment is based mainly on the use of anti‐fibrotic drugs, such as pirfenidone and nintedanib, which are partially effective (Lederer & Martinez, 2018). Unfortunately, to date, lung transplantation is often the only viable therapeutic approach and no therapy has proven effective in prolonging survival (Hennion et al., 2022). Therefore, new therapeutic molecules with the same properties as synthetic drugs, better efficacy and tolerability, and fewer side effects on PF were needed.

In this regard, in our laboratory, the beneficial actions of natural bioactive phytocompounds, such as extracts and fixed or essential oils, on the prevention/treatment of PF induced by bleomycin have been published, such as *Pistacia lentiscus* and *Olea europaea* (Abidi et al., 2017; Bahri et al., 2023). Some of these studies have shown that phenolic compounds, such as flavonoids, phenolic acids, and tannins, could play a crucial role in the management of this respiratory pathology.

This study provides the first evidence of the beneficial effects of *Eucalyptus cinerea* seeds aqueous extract against bleomycin‐induced pulmonary fibrosis, oxidative stress, metabolic and hematological alterations. Additionally, this investigation aimed to characterize the functional foods, assess its antioxidant properties, and elucidate its in vivo effects on inflammatory biomarkers, antioxidant activities, and pulmonary integrity.

## 2 MATERIALS AND METHODS

### 2.1 Ethical approval

Experimental protocols were used in accordance with Tunis University's local ethics committee (University of Jendouba: UJ2021‐03‐4022) in accordance with the International Council of Laboratory Animal Science for the use and care of animals. The protocol was approved by the “Bio‐medical Ethics Committee (CEBM)” of the Pasteur Institute of Tunis, published in JORT47‐2001.

### 2.2 Animals and treatment

Healthy adult male Wistar rats (210–240 g) were purchased from the Society of Pharmaceutical Industries of Tunisia: SIPHAT (Tunisia). The animals were separated into different groups and acclimatized for 2 weeks with a standard pellet diet (standard pellet diet Badr‐ Utique‐TN) and water ad libitum (22°C ± 2°C; 12 h dark/light cycle). Animals were used in accordance with Tunis University's local ethics committee (University of Jendouba: UJ2021‐03‐4022) in accordance with the International Council of Laboratory Animal Science for the use and care of animals. The protocol was approved by the “Bio‐medical Ethics Committee (CEBM)” of the Pasteur Institute of Tunis, published in JORT47‐2001.

#### 2.2.1 Induction of experimental pulmonary fibrosis in rats

For induction of pulmonary fibrosis, all rats underwent anesthesia by intraperitoneal injection of 75 mg/kg bw of pentobarbital sodium solution. Each anesthetized rat was immediately suspended from gallows. Induction of fibrosis was done by an intra‐tracheal instillation of 2 mg/kg bw of BLEO (Bleomycin®, Laboratories Aventis, France) in 200 μL saline (0.9%).

Conecrning the experimental design, a total of thirty rats were randomly divided into five experimental groups of six animals each (*n* = 6).

Group I (Normal control): rats received any treatment, only 0.9% saline (orally 5 mL/kg bw); Group II (BLEO model group): rats received a single intratracheal instillation of bleomycin (2 mg/kg b.w.), 3 days later they received by gavage 0.9% saline (5 mL/kg bw) once daily for 21 days.

Group III (BLEO + ECAE‐50): rats received BLEO solution intratracheally (2 mg/kg bw); 3 days later they were treated with ECAE (50 mg/kg) for 21 days.

Group IV (BLEO + ECAE‐100): Rats received BLEO solution intratracheally (2 mg/kg bw); 3 days later they were treated with ECAE (100 mg/kg) daily for 21 days.

Group V (BLEO + GA‐50): Rats received BLEO solution intratracheally (2 mg/kg bw); 3 days later, they were treated with Gallic acid (50 mg/kg) daily for 21 days.

For treatment, gastric gavage was daily realized. During the experiment, the body weight of rats in each group was measured on days 1, 5, 10, and 21.

#### 2.2.2 Organ sampling and broncho‐alveolar lavage fluid (BaLF)

At the end of the experimental period, animals were anesthetized with ketamine (80 mg/kg bw, intraperitonial injection) and sacrificed by decapitation. Blood samples for biochemical analyses were taken in heparinized tubes and centrifuged at 3000 × *g* for 15 min.

Sections of the diaphragm and anterior thorax allowed us to extract the lungs and were then quickly weighed. Same samples were homogenized (1:2, w/v) in 50 mM Tris buffer (pH = 7.4) containing 150 mM of NaCl using an Ultra‐Tunax. Homogenates were centrifuged at 5000 × *g* for 25 min at 4°C, and aliquots of supernatant were stored at −80°C until use. Before the extraction of the heart‐lung block, the trachea was exposed and cannulated. Intratracheal injection of saline (4 × 5 mL) was performed via this catheter, and the liquid was sucked back by gentle suction injected between two fractions: bronchoalveolar lavage fluid (BaLF). It was then centrifuged at 250 × *g* for 10 min to exclude cells and waste that may be present in the sample.

The lobes of left lungs were fixed by intratracheal injection of a 10% formalin solution (6–8 mL) and immersed in formalin for 48 h before histological examination. Then, the remaining lungs were rapidly excised, trimmed of extraneous tissue, rinsed, and weighed for the relative lung weight determination.

### 2.3 Seeds collection and *Eucalyptus cinerea* aqueous extract (ECAE) preparation

The seeds of *Eucalyptus cinerea* F. Muell. ex Benth were collected in November 2024 near the Zarga region (Ain Draham, Tunisia). The specimens (No. EC201) were then deposited with the herbarium of the Higher Institute of Biotechnology of Béja, University of Jendouba. After collection, the seeds were rinsed with distilled water to eliminate contaminants. The seeds were then homogenized and dried in an incubator at 50°C for 72 h and powdered in an electric blender. For the preparation of seed extracts, many test portions of 1 g of powder were freshly dissolved in double distilled water (1/20; w/v) and filtered through a colander (0.5 mm mesh size). The samples were centrifuged at 5000 rpm for 10 min, and the supernatants were recovered and lyophilized. Finally, the residues thus obtained were weighed, and the products were stored at −40°C until use.

For animal treatment, freshly prepared doses were administered daily to rats by gavage throughout the experiment by introducing the resulting lyophilized extract into distilled water. Concentrations underwent quality control by quantifying total polyphenols and flavonoids, coupled with in vitro antioxidant capacity assessment using the DPPH (2,2‐diphenyl‐1‐picrylhydrazyl) assay. These investigations are considered a reliable and rapid reference method for confirming the stability of bioactive compounds at the selected extract concentrations.

#### 2.3.1 High‐performance liquid chromatography‐mass spectrometry identification of phenolic compounds in ECAE

The identification of phenolic compounds in *Eucalyptus cinerea* seeds aqueous extract (ECAE) was realized using HPLC–MS technique. The analytical process using a Shimadzu Nexera X2 HPLC system (Kyoto, Japan) instrument for rapid and reliable separation and purification, equipped with a Shim‐pack GIST C18 column (4.6 × 150 mm, 3 μm). The mobile phase checked of water with 0.1% formic acid (A) and acetonitrile with 0.1% formic acid (B), with a gradient elution of 10%–90% B over 25 min at a flow rate of 0.5 mL/min. A 10 μL injection volume was used with a constant column temperature of 40°C. Detection was performed using Multiple Reaction Monitoring (MRM) on a Shimadzu LCMS‐8045 triple quadrupole mass spectrometer. Samples were ionized in negative electrospray mode under the following optimized conditions: capillary voltage 3.2 kV, desolvation temperature 350°C, nebulizer gas flow 2.5 L/min, and collision energy between 15 and 35 eV depending on the target compound. Calibration curves were prepared using authentic standards available in our laboratory such as quercetin (PubChem CID: 5280343), rutin (PubChem CID: 5280805), kaempferol (PubChem CID: 5280863), apigenin (PubChem CID: 5280443), luteolin (PubChem CID: 5280445), chlorogenic acid (PubChem CID: 1794427), ferulic acid (PubChem CID: 445858), syringic acid (PubChem CID: 10742), and the results were expressed as mg/g of dry extract. Finally, data processing was performed using LabSolutions LC–MS software (Shimadzu).

#### 2.3.2 In vitro antioxidant capacities

The capacities of ECAE to scavenge hydrogen peroxide (H_2_O_2_) were evaluated according to the experience previously described (Bhardwaj et al., 2022). Briefly, 0.1 mL of ECAE (10–550 μg/mL) was added to made volume up to 0.4 mL with the addition of 50 mM (pH 7.4) phosphate buffer and (2 mM) H_2_O_2_ (PubChem CID: 784) solution (0.6 mL). The product was vortexed and kept for 10 min, and then, the absorbance was measured at 230 nm. Gallic acid was used as a reference antioxidant molecule. The ability of the extracts to scavenge H_2_O_2_ was assessed using the following equation.

$$\begin{array}{c} {\text{H}}_{2}{\text{O}}_{2}\;\text{scavenging activity percentage}\;\left(\%\right)\\ =\frac{\mathrm{Abs}\;\text{blank}-\mathrm{Abs}\;\text{Sample}}{\mathrm{Abs}\;\text{blank}}\times 100 \end{array}$$

DPPH^•^ (PubChem CID: 5360881) was also used to determine the free radical scavenging activity of the ECAE, according to the method previously described (Elfalleh et al., 2009). Briefly, 500 μL at different increasing concentrations of extract (from 10 to 400 μg/mL) were prepared, and each concentration was added to 375 μL of an ethanolic solution of 2,2‐diphenyl‐1‐picrylhydrazyl (DPPH^**•**^, 96%). The mixture was kept in the dark at room temperature for 60 min. Absorbance was recorded at 517 nm. Finally, the percentage inhibition of free radical DPPH^**•**^ was calculated, and the IC_50_ value was determined from the graph curve. Ascorbic acid (PubChem CID: 54670067) was used as a reference antioxidant molecule. The ability of the extracts to scavenge DPPH^**•**^ was determined using the following equation:

$$\begin{array}{c} \text{DPPH scavenging activity percentage}\;\left(\%\right)\\ =\frac{\mathrm{Abs}\;\text{blank}-\mathrm{Abs}\;\text{Sample}}{\mathrm{Abs}\;\text{blank}}\times 100 \end{array}$$

### 2.4 Determination of relative lung weight

The animals' body weights were recorded on the day of euthanization. The weight of the lungs was measured after careful harvesting, squeezing out blood, blotting on the filter paper, and washing in ice‐cold saline. A digital weighing balance was used. Relative lung weight was computed using the following formula:

$$\begin{array}{c} \text{Relative organ to body weight}\;\left(\%\right)\\ =\frac{\text{Individual lung weight}\;\left(\text{grams}\right)}{\text{Body weight of individual}\;\mathrm{rat}\;\mathrm{on}\;\mathrm{day}\;\text{of sacrifice}\;\left(\text{grams}\right)}\times 100 \end{array}$$

### 2.5 Histological study

For histological analysis, the lungs were fixed in 10% formalin solution for 24 h, dehydrated in a graded series of ethanol, embedded in paraffin, cut into 5 mm thick serial sections, and stained with hematoxylin and eosin (H&E) to identify inflammatory cells. The entire lung section was observed at ×100 magnification under light microscope. We took four sections per lung and for the precise evaluation of the Ashcroft score; we analyzed 4 histological sections per animal, distributed throughout the lung. On each section, we evaluated at least 10 randomly selected microscopic fields. The evaluation of fibrosis score was carried out using a blinded semi‐quantitative system to assess the extent and severity of fibrosis in the lung parenchyma. The extent of pulmonary fibrosis was evaluated using a semi‐quantitative Ashcroft scoring system, adapted from previously described methods (Ashcroft et al., 1988; Hubner et al., 2008). This scale ranges from 0 to 8, reflecting the progressive severity of fibrotic alterations in lung tissue. A score of 0 corresponds to normal lung histology, while grade 1 indicates minimal fibrous thickening of the alveolar or bronchial walls. Grades 2 and 3 represent moderate thickening without significant disruption of the overall lung architecture. Scores of 4 and 5 are assigned when fibrosis becomes more pronounced, with clear structural damage and the appearance of fibrous bands or small fibrotic masses. Grades 6 and 7 reflect advanced fibrosis characterized by severe architectural distortion, extensive fibrotic areas, and the presence of honeycomb‐like structures. Finally, grade 8 corresponds to complete fibrotic obliteration of the observed field.

### 2.6 Broncho‐alveolar lavage

The bronchoalveolar lavage fluid (BaLF) sample was centrifuged at 3000 × *g* for 5 min at 4°C. Afterwards, the supernatant was removed, and the cell pellet was resuspended with 50 μL of saline solution. 10 μL of the cell suspension was pipetted, and the total cell number was counted using a hemocytometer. 30 μL of cell suspension was then pipetted, and cell smears were prepared and stained with Wright's Giemsa to distinguish different cell types (multi‐lobed nuclei or mononuclear cells) under a light microscope, type Euromex, and a 100 × oil immersion objective was used.

### 2.7 Assessment of pulmonary inflammatory/immunology status

#### 2.7.1 Determination of CRP level and alkaline phosphatase activities

Plasmatic C‐reactive protein (CRP, Ref 45,027) levels and plasmatic/pulmonary alkaline phosphatase (ALP, Ref 13,026) activities were estimated using commercially available diagnostic kits (Biomaghreb, Ariana, TN, ISO 9001 certificate).

#### 2.7.2 Immunological parameters evaluation

To evaluate the curative actions of ECAE on BLEO‐induced pulmonary fibrosis, the analysis of red blood and white blood cell (WBC) subtypes including lymphocytes, monocytes, neutrophils, and eosinophils was performed to assess toxic stress induced by bleomycin. These parameters were determined using a MYTHIC 22 automatic hematology analyzer.

### 2.8 Assessment of oxidative stress and antioxidants biomarkers

Pulmonary and plasmatic oxidative stress and antioxidant biomarkers were determined by evaluation of protein contents, malondialdehyde (MDA), thiol group, and assessment of antioxidant enzyme activities: superoxide dismutase (SOD), catalase (CAT), and glutathione peroxidase (GPx) and non‐enzymatic antioxidant levels: thiol (–SH) groups and reduced glutathione (GSH).

The lung and plasma total protein concentrations were determined using commercially available diagnostic kits (Biomaghreb, Tunisia).

The lung and plasma lipid peroxidation were carried out by the MDA determination (Draper & Hadley, 1990). The homogenates of the lung and plasma were added to a BHT (PubChem CID: 31404)‐TCA (PubChem CID: 6421) solution (1% BHT (w/v) dissolved in 20% TCA (w/v)) and centrifuged at 1000 × g for 5 min. The obtained supernatant was mixed with a buffered solution composed of 120 mM of TBA (PubChem CID: 2723628), Tris (26 mM), and HCl (0.5 N). The product was heated at 80°C for 10 min. The results were determined at 532 nm, and MDA levels were measured using an extinction coefficient for MDA‐TBA of 1.56 × 105 M^−1^ cm^−1^.

The thiol groups (‐SH) content in lungs and plasma was determined, as previously described (Ellman, 1959). The concentration of thiol groups was calculated by subtraction operation between two absorbances (A2 and A1) using a molar extinction coefficient of 13.6 × 103 M^−1^ cm^−1^. The results were expressed in nmol of thiol groups/mg proteins.

For pulmonary and plasmatic reduced glutathione (GSH), 500 μL of each homogenate were added to 20 mM, pH 4.7 of EDTA (PubChem CID: 6049), 400 μL of cold distilled water, and mixed with 100 μL of TCA (50%). The mixtures were stirred and centrifuged at 1200 × *g* for 15 min. The 2 mL of recovered supernatant was blended with 400 μL Tris buffer (0.4 mM, pH 8.9) and 10 μL of 0.01 M DTNB (PubChem CID: 6254). Finally, the absorbance was read at 412 nm (Sedlak & Lindsay, 1968).

The spectrophotometric method was used to evaluate the SOD activities in lungs and plasma using the epinephrine (PubChem CID: 5816)/adenochrome (PubChem CID: 5898) system and using bovine catalase (PubChem CID: 135337101) (CAT, 0.4 U/mL) and epinephrine (5 mg/mL) as enzymes. Changes in absorbance were evaluated at 480 nm (Kakkar et al., 1984).

The activity of CAT was assessed by measuring the rate of hydrogen peroxide disappearance by spectrophotometry at 240 nm, which will be degraded into H_2_O and O_2_. CAT activity is expressed in μmoles H_2_O_2_/mg protein (Aebi, 1984).

The assessment of GPx pulmonary and plasma activities was determined according to the method previously described (Flohé & Günzler, 1984).

### 2.9 Pulmonary and plasma intracellular mediators

The pulmonary and plasma hydrogen peroxide (H_2_O_2_) levels were determined as follow. Briefly, the hydrogen peroxide reacts with p‐hydroxybenzoic acid and 4 aminoantipyrine in the presence of peroxidase leading to the formation of quinoneimine that has a pink color detected at 505 nm (Dingeon et al., 1990).

The levels of calcium (Ca, Ref 01023) in lungs and plasma were measured colorimetrically using commercially available diagnostic kits (Biomaghreb, Ariana, TN, Tunisia).

### 2.10 Plasmatic electrolytes levels

The protective potential of ECLAE against BLEO was determined by the quantitative dosage of certain electrolytes in the plasma of rats: sodium (Na, Ref: 14101), potassium (K, Ref: 14102), and magnesium (Mg, Ref: 14103), using diagnostic kits obtained from the company Biomaghreb (Ariana, TN).

### 2.11 Statistical analysis

All experimental data were analyzed using the GraphPad Prism 8.0.2 software (San Diego, CA, USA) and are expressed as the mean ± standard deviation (SD). The data represent observations from 6 samples. Statistical differences between groups were assessed using a one‐way analysis of variance (ANOVA). The difference is considered significant when the *p* < 0.05.

## 3 RESULTS

### 3.1 Identification of phenolic compounds by HPLC–MS and in vitro antioxidant capacities

**FIGURE 1:**
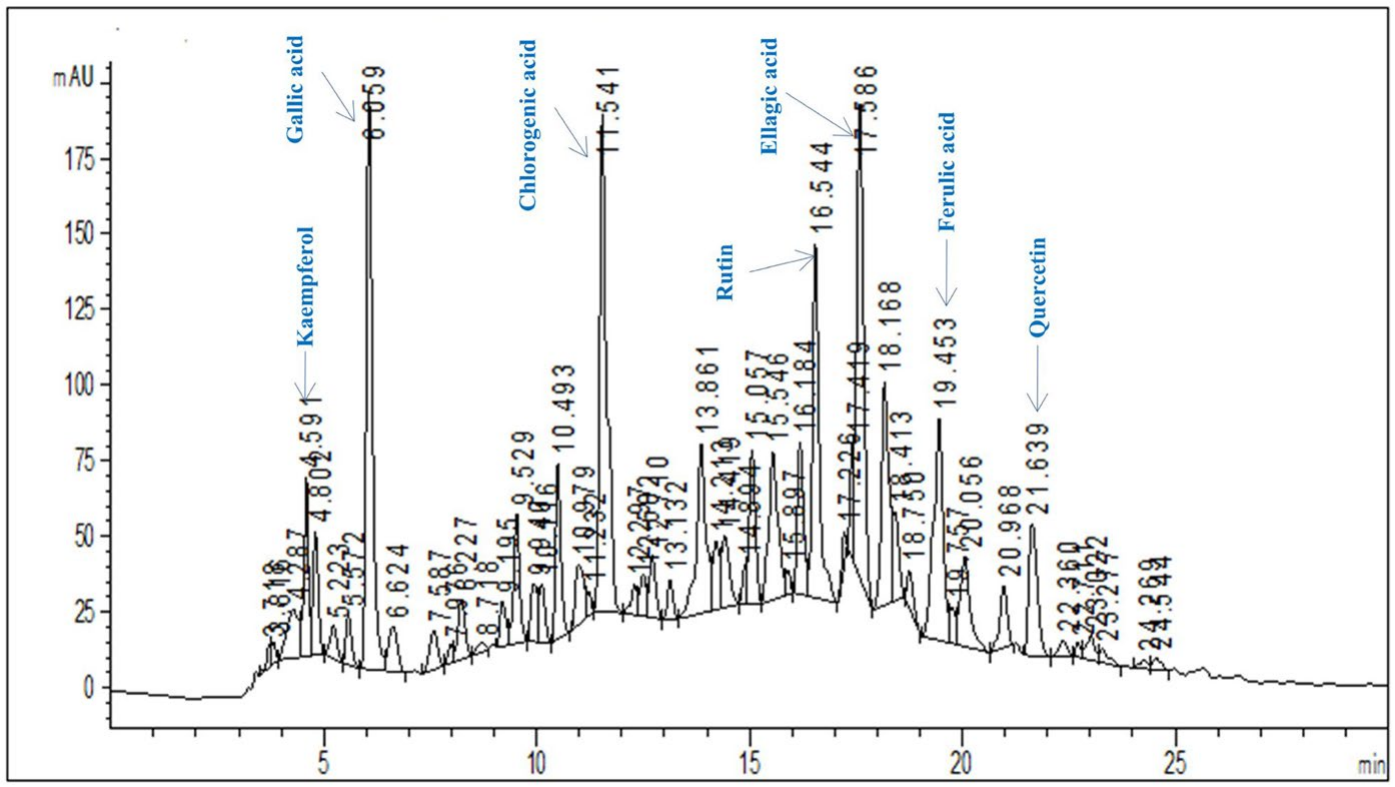
Chromatographic profiles of *Eucalyptus cinerea* seeds aqueous extract (ECAE). Peaks assignments are given in Table 1.

The LC–MS technique allowed us to identify seven phenolic compounds in ECAE. These compounds belong to three main classes of polyphenols: phenolic acids, flavonoids, and tannins. Among the phenolic acids found in ECAE, we identified gallic, chlorogenic, and ferulic acids. Three flavonoids were also detected in our extract: kaempferol, rutin, and quercetin. Finally, ellagic acid, a tannin, has been isolated from the seeds of *Eucalyptus cinerea* (Table 1 and Figure 1).

**TABLE 1:** High‐performance liquid chromatography‐mass spectrometry (HPLC‐MS) identification of phenolic compounds in *Eucalyptus cinerea* seeds aqueous extract (ECAE).

Peak N°	Retention time (min)	Area	Molecular formula	Tentative identification	Concentration (μg/g extract)
4	4.591	439.35	C_15_H_10_O_6_	Kaempferol	0.19
8	6.0586	1944.23	C_7_H_6_O_5_	Gallic acid	0.87
21	11.540	1952.75	C_16_H_18_O_9_	Chlorogenic acid	2.16
34	16.544	1274.12	C_27_H_30_O_16_	Rutin	1.41
37	17.586	1765.13	C_14_H_6_O_8_	Ellagic acid	1.97
41	19.452	1158.69	C_10_H_10_O_4_	Ferulic acid	0.56
45	21.639	611.89	C_15_H_10_O_7_	Quercetin	0.37

**FIGURE 2:**
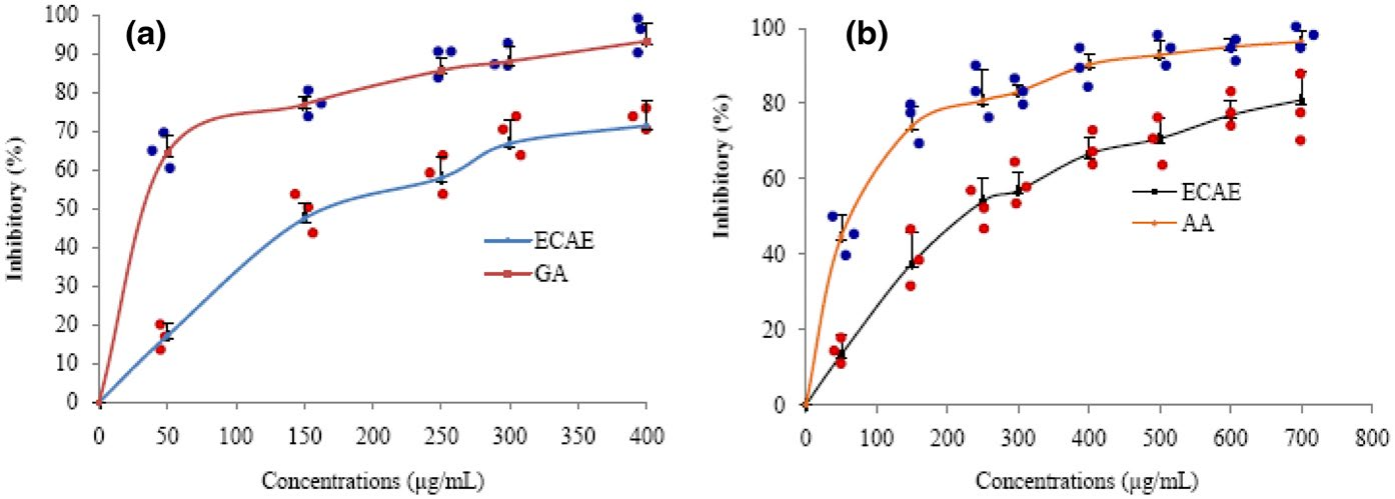
Antioxidant activities of *Eucalyptus cinerea* seeds aqueous extract (ECAE); (a) H_2_O_2_ scavenging activity, (b) DPPH^•^ radical scavenging activity; GA, gallic acid; AA, ascorbic acid. All samples were analyzed in three replicates. Data are expressed as means ± SD.

Concerning the H_2_O_2_ and/or DPPH^•^ radical scavenging activities of ECAE, the present results showed significant differences (*p* < 0.05) between the two tests examined (Figure 2). Indeed, ECAE showed the best antioxidant activity (IC_50_ = 96.91 μg/mL) against DPPH^•^ compared to that obtained from H_2_O_2_ activity (IC_50_ = 321.89 μg/mL). In addition, the IC_50_ of ECAE was significantly higher than those of gallic acid (IC_50_ = 33.64 μg/mL) and ascorbic acid (12.64 μg/mL), used as reference antioxidant molecules (Figure 2).

### 3.2 Effect of ECAE and BLEO on relative lung weight, food consumption and water intake

**FIGURE 3:**
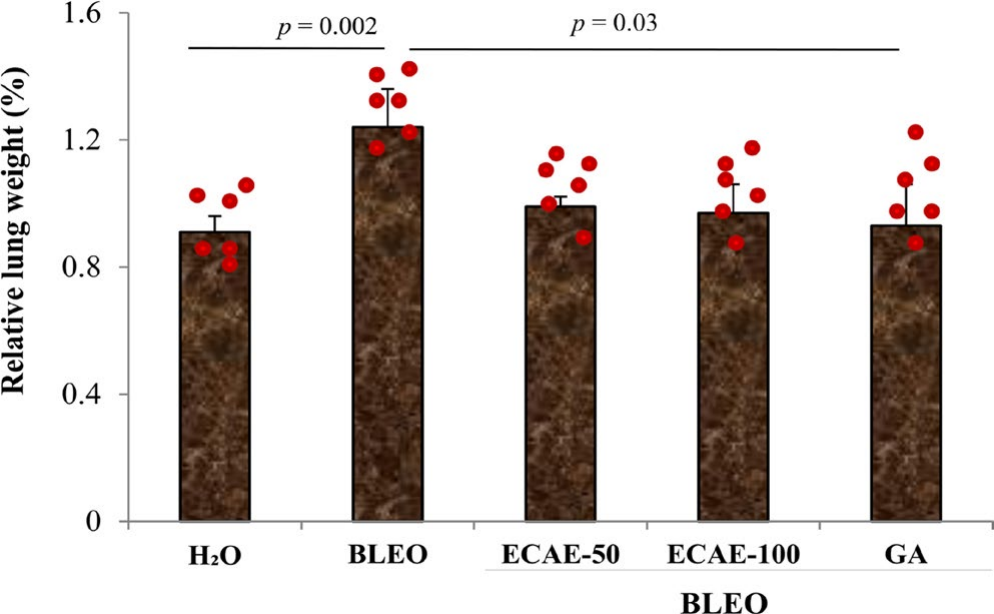
Effects of *Eucalyptus cinerea* seeds aqueous extract (ECAE) and gallic acid (GA) on relative lung weight in rats induced with bleomycin (BLEO). H_2_O: Normal control group, BLEO: Intratracheal instilled bleomycin group, ECAE at different doses (50 and 100 mg/kg) after BLEO instillation. Data are expressed as means ± SD (*n* = 6). Statistical differences are indicated by exact *p*‐values between groups (ANOVA test followed by post hoc test).

Generally, the present research showed that the ECAE significantly reduced (*p* < 0.05) relative lung weight in bleomycin (BLEO) rats compared with the weights in the negative control rats (Figure 3). It was also noted that *Eucalyptus cinerea* extract at 100 mg Kg^−1^ bw and gallic acid (50 mg Kg^−1^ bw) reduced the relative lung weight of BLEO rats to normal (Figure 3).

Changes in food and water consumption in the different groups were determined between days 1 and 21. Indedd, BLEO caused a significant decrease of food consumption (*p* < 0.05) compared to the control group throughout the 21‐day experiment. However, the ECAE or GA groups showed a significant (*p* < 0.05) dose‐dependent increase of this parameter (Table 2). Suggesting that the increase in body weight gain was primarily related to the appetitive‐stimulating effect of ECAE.

**TABLE 2:** Effects of bleomycin (BLEO) instillation and *Eucalyptus cinerea* seeds aqueous extract (ECAE)/gallic acid (GA) treatments on food and water consumption in rats.

Group		Day 1	Day 5	Day 10	Day 21
H_2_O	Food (g)	19.83 ± 1.47	19.33 ± 1.75	20.17 ± 1.37	20.25 ± 1.57
	Water (mL)	37.95 ± 1.56	39.02 ± 2.15	41.27 ± 1.70	43.23 ± 1.97
BLEO	Food (g)	6.51 ± 1.04*	7.19 ± 0.86*	7.53 ± 0.55*	8.85 ± 0.88*
	Water (mL)	22.08 ± 2.87*	20.75 ± 1.33*	19.35 ± 1.65*	18.66 ± 1.93*
ECAE‐50	Food (g)	8.03 ± 1.28#	8.17 ± 1.03#	10.02 ± 1.12#	14.30 ± 1.15#
	Water (mL)	24.71 ± 1.69#	26.86 ± 3.08#	27.99 ± 1.54#	30.59 ± 1.94#
ECAE‐100	Food (g)	12.83 ± 1.62#	12.55 ± 1.69#	13.07 ± 1.41#	17.03 ± 1.03#
	Water (mL)	30.27 ± 2.82#	32.38 ± 1.85#	33.78 ± 2.64#	38.25 ± 3.18#
GA‐50	Food (g)	12.43 ± 2.41#	11.93 ± 0.71#	12.62 ± 0.89#	16.68 ± 1.27#
	Water (mL)	28.42 ± 3.97#	31.80 ± 3.27#	32.10 ± 2.93#	35.26 ± 1.98#

*Note*: H_2_O: Normal control group; BLEO: Intratracheal instilled bleomycin group; ECAE: *Eucalyptus cinerea* seeds aqueous extract at different doses (50 and 100 mg/kg) after BLEO instillation. Data are expressed as means ± SD (*n* = 6); *p < 0.05 compared to control group and #*p* < 0.05 compared to BLEO group (ANOVA test).

The results of the variation in water consumption were recorded throughout the treatment period in Table 2. BLEO caused a significantly reduced access to this parameter compared to the normal control group. However, treatment with ECAE and GA significantly increased access to water consumption, dose‐dependently, compared to the BLEO group.

### 3.3 Effects of ECAE, GA and BLEO on histopathological structure of pulmonary

**FIGURE 4:**
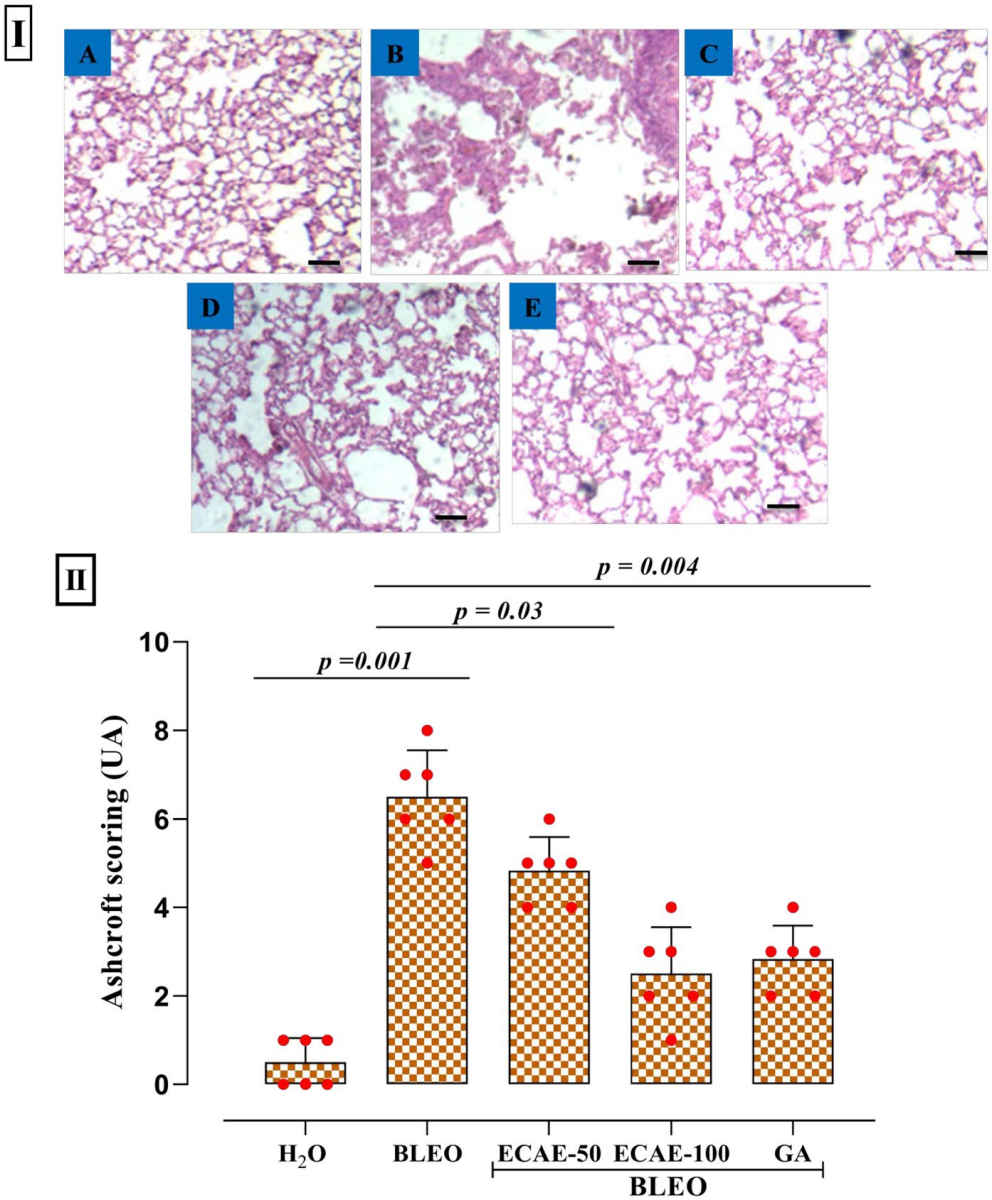
Pulmonary histological observation (I) and Fibrosis score (II), showing the protective action of *Eucalyptus cinerea* seeds aqueous extract (ECAE, 50 and 100 mg/kg, b.w.) and gallic acid (GA, 50 mg/kg, b.w.) against Bleomycin (BLEO) intoxication. Animals were treated with two doses of ECAE (50 and 100 mg/kg, b.w., p.o.) or vehicle (NaCl 0.9%). (a) H_2_O + NaCl; (b) BLEO group; (c, d) BLEO + ECAE (50 and 100 mg/kg, b.w., p.o., respectively); (e) BLEO + GA (50 mg/kg, b.w., p.o), H&E, ×200, scale bar = 20 μm.

Generally, control lungs showed normal pulmonary architecture with normal alveolar spaces and normal thickening of alveolar septa (Figure 4a). Whereas, lungs from rats intoxicated with BLEO alone were characterized by lymphocytic inflammatory redesign, disturbance of the cellular architecture with fibrous alterations as well as the presence of small nodules. The lungs of this group are characterized by a pronounced inflammatory reaction with massive infiltration of inflammatory cells, alveolar wall thickening, and the presence of inter‐ and intra‐alveolar plasma cells (Figure 4b). However, treatments with various doses of ECAE (50 and 100 mg/kg, b.w.) or GA (50 mg/kg, b.w.) reduced the severity almost all BLEO‐induced lung damages (Figure 4c–e). Most importantly, the BLEO/ECAE‐100 mg/kg group showed minimal wall thickening and no detectable damage to lung architecture (Figure 4d).

The severity of fibrosis was assessed using a semi‐quantitative Ashcroft scoring system. Drastically, a significant increase in the fibrosis score was observed in the BLEO group compared to the normal control group. Conversely, in the ECAE and GA treated groups, this score decreased significantly compared to the BLEO group (Figure 4).

### 3.4 Impacts of ECAE/GA on bronchoalveolar fluid cells and lung inflammation markers

To evaluate the protective effect of ECAE on BLEO‐induced pulmonary fibrosis in rats, the number of bronchoalveolar lavage (BaLF) cells and biomarkers of inflammation were estimated.

**FIGURE 5:**
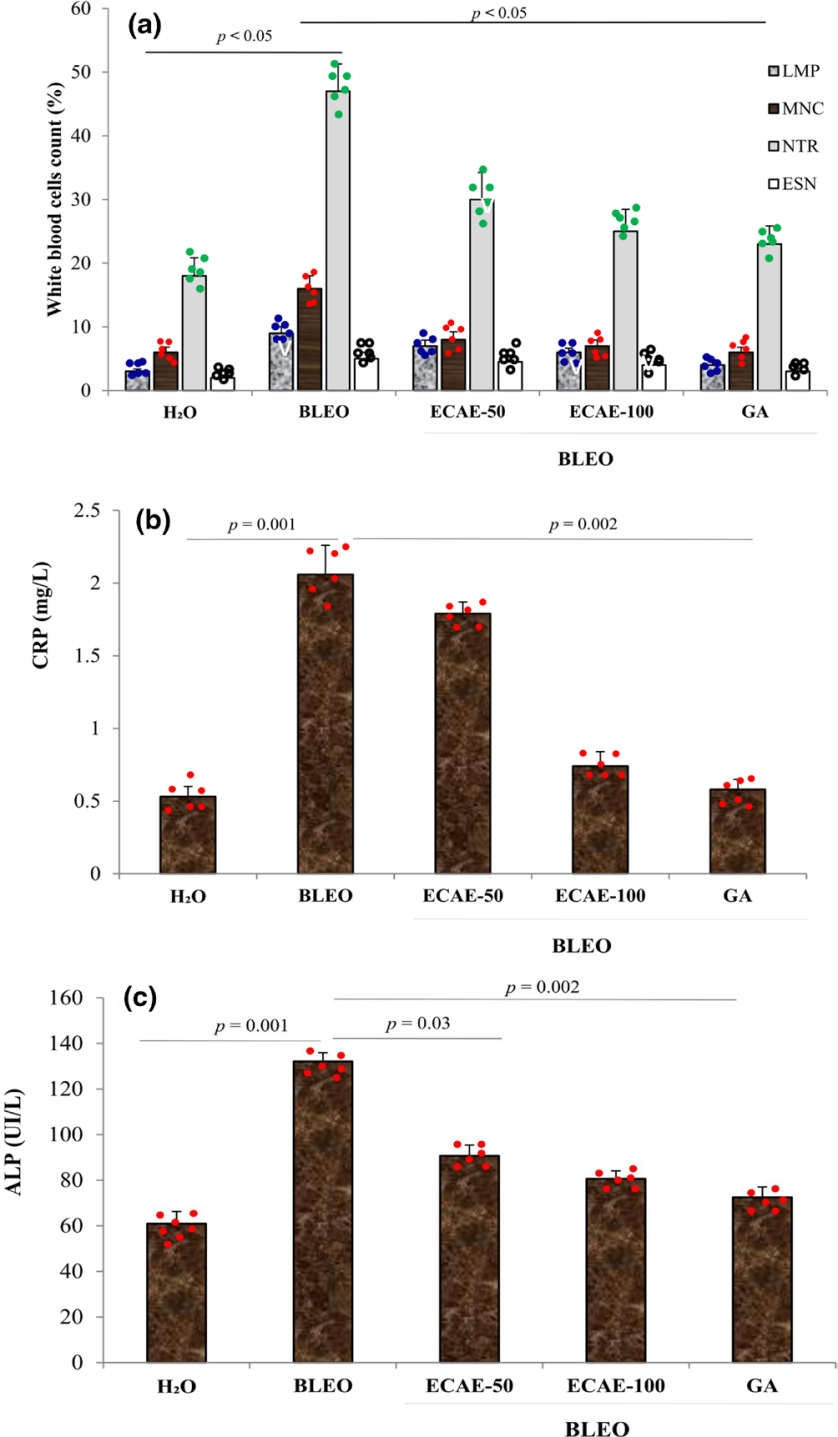
Effects of *Eucalyptus cinerea* seeds aqueous extract (ECAE, 50 and 100 mg/kg, b.w.) and gallic acid (GA, 50 mg/kg, b.w.) on immune cells in broncho‐alveolar lavage fluid (a) and inflammatory markers: CRP (b) and ALP (c) during bleomycin (BLEO, 2 mg/kg b.w.)‐induced pulmonary fibrosis. Data are expressed as means ± SD (*n* = 6). Statistical differences are indicated by exact *p*‐values between groups (ANOVA test followed by post hoc test). ALP, alkaline phosphatase; CRP, C‐reactive protein; LMP, lymphocyte; MNC, monocyte; NTR, neutrophil; ESN, eosinophil.

As shown in Figure 5a, the hematological parameters exhibited significant alterations following intoxication with BLEO. This product resulted in marked changes in blood cell counts, indicating potential adverse effects on blood composition and overall health. Treatment with ECAE effectively normalized these parameters, bringing them closer to control levels. Similarly, GA administration also resulted in notable improvements in hematological parameters compared to the BLEO‐treated group. More importantly, this observation highlights the promising therapeutic potential of ECAE and GA in reversing hematological disturbances caused by BLEO, offering valuable insights into their effectiveness in managing lungs and inflammatory conditions.

The evaluation of CRP and ALP levels revealed significant findings regarding inflammation and lung status in the treatment groups (Figure 5). The intratracheal instilled bleomycin resulted in a notable increase in CRP level and ALP activity, indicating heightened inflammatory responses. On the other hand, treatment with ECAE led to a significant reduction in both CRP contents and ALP activities, suggesting its potential anti‐inflammatory properties (Figure 5b,c).

### 3.5 ECAE and GA attenuates redox status damages in BLEO‐induced pulmonary fibrosis

#### 3.5.1 Effects of ECAE/GA on lipoperoxidation levels increase

**FIGURE 6:**
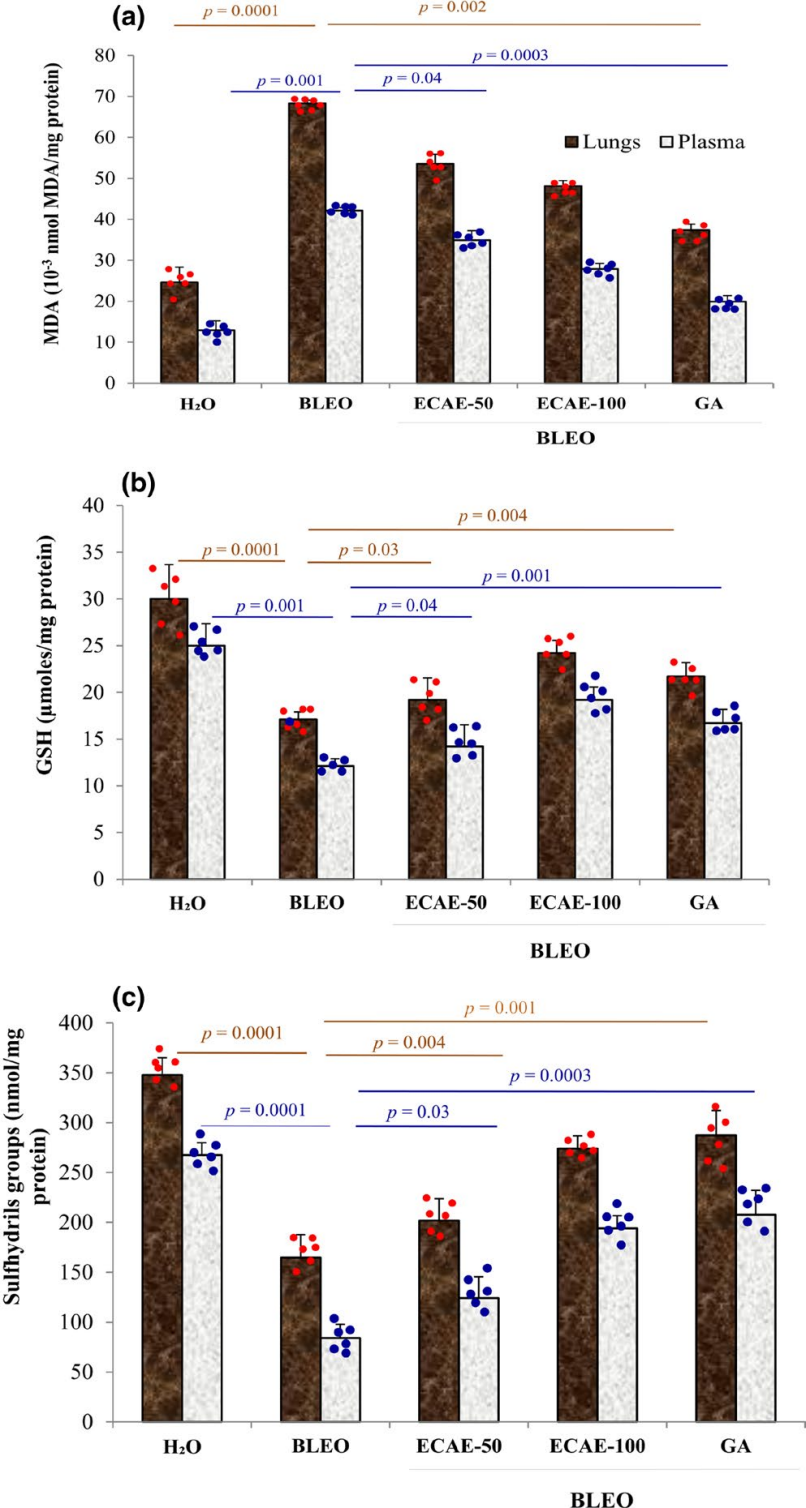
Effects of ECAE/GA and BLEO on pulmonary and plasmatic malondialdehyde (MDA, a) content and non‐enzymatic antioxidants: Reduced glutathione (GSH, b) and sulfhydryl groups (‐SH, c) levels. Data are expressed as means ± SD (*n* = 6). Statistical differences are indicated by exact *p*‐values between groups (ANOVA test followed by post hoc test). BLEO, Bleomycin; ECAE, *Eucalyptus cinerea* seeds aqueous extract.

For further investigation of the protective effect of ECAE on BLEO‐induced pulmonary fibrosis in rats, the lung and plasmatic MDA levels were determined among groups. Induction of fibrosis by BLEO causes a significant increase (*p* < 0.05) of MDA content in the lung and plasma when compared to the control group. However, consumption of the *Eucalyptus* seeds aqueous extract significantly and dose‐dependently decreased pulmonary and plasmatic MDA content in inflamed rats (Figure 6a).

#### 3.5.2 Pulmonary and plasmatic GSH and ‐SH contents

We also investigated the levels of non‐enzymatic antioxidants in lungs and plasma. The intoxication with BLEO exerted a significant (*p* < 0.05) decrease of reduced glutathione (GSH) and sulfhydrils groups (‐SH) levels (Figure 6b,c, repectiveley). In contrast, subacute treatment with ECAE (50 and 100 mg/kg, b.w.) or GA (50 mg/kg, b.w.) provided significant and dose‐dependent improvement against depletion induced by BLEO.

#### 3.5.3 Effect of ECLAE on BLEO‐induced antioxidant enzyme activities

**FIGURE 7:**
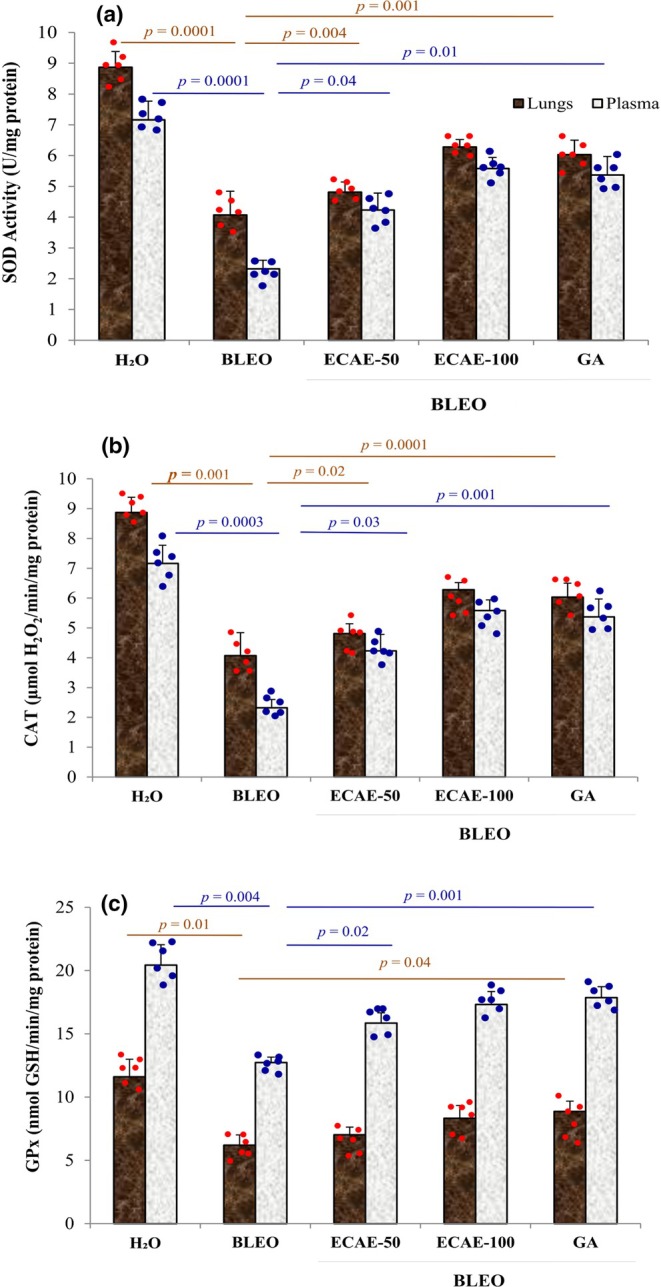
Effects of ECAE and GA on antioxidant enzymes: SOD (a), CAT (b) and GPx (c) during BLEO‐induced pulmonary fibrosis during three weeks. Data are expressed as means ± SD (*n* = 6). Statistical differences are indicated by exact *p*‐values between groups (ANOVA test followed by post hoc test). BLEO, Bleomycin; CAT, Catalase; ECAE, *Eucalyptus cinerea* seeds aqueous extract; GA: Gallic acid; GPx, glutathione peroxidase; SOD, Superoxyde dismutase.

The antioxidant enzyme activities recorded show that the SOD, CAT, and GPx are significantly decreased (*p* < 0.05) in the fibrosis group (BLEO) compared to the control. The treatment of rats with ECAE significantly increased BLEO‐induced reduction of antioxidant enzyme activities to near control levels with the highest dose (Figure 7).

### 3.6 Effects of ECAE, GA on BLEO‐induced intracellular mediators deregulation

BLEO exposure significantly disrupted the intracellular mediators in both lungs and plasma, as evidenced by a marked increase in the levels of H_2_O_2_ and Ca, compared to control animals as shown in Table 3. Treatment with the ECAE restored the levels of both H_2_O_2_ and Ca^2+^ toward normal levels (*p* < 0.05 versus BLEO group). This indicates that ECAE/GA effectively alleviated the cytotoxic effects associated with BLEO, thereby restoring normal intracellular mediator levels. Interestingly, the restoration of Ca^2+^ ions to a level close to normal control group confirms the bronchodilator role of *Eucalyptus cinerea* seeds.

**TABLE 3:** Effect of bleomycin (BLEO) instillation and *Eucalyptus cinerea* seeds aqueous extract (ECAE)/gallic acid (GA) on bleomycin (BLEO)‐induced changes in lungs and plasma hydrogen peroxide (H_2_O_2_) and calcium levels.

Group	H_2_O_2_ (nmol/mg protein)		Calcium (mmol/mg protein)	
	Lung	Plasma	Lung	Plasma
H_2_O	15.95 ± 1.65	3.62 ± 0.08	4.72 ± 0.17	3.23 ± 0.37
BLEO	38. 82 ± 3.69*	12.58 ± 2.06*	9.53 ± 0.56*	8.66 ± 1.06*
ECAE‐50	36.89 ± 3.24	8.44 ± 0.86#	7.29 ± 0.43#	3.59 ± 0.14#
ECAE‐100	20.81 ± 2.68#	5.64 ± 0.30#	3.87 ± 0.24#	3.25 ± 0.23#
GA	24.76 ± 1.74#	6.82 ± 0.25#	3.10 ± 0.03#	4.26 ± 0.91#

*Note*: H_2_O: Normal control group; BLEO: Intratracheal instilled bleomycin group; ECAE: *Eucalyptus cinerea* seeds aqueous extract at different doses (50 and 100 mg/kg) after BLEO instillation. Data are expressed as means ± SD (*n* = 6); *p < 0.05 compared to control group and #*p* < 0.05 compared to BLEO group (ANOVA test).

### 3.7 Beneficial impacts of ECAE and GA against BLEO‐induced electrolyte changes

The actions of ECAE/GA treatments on plasmatic electrolytes in rats with BLEO‐induced pulmonary fibrosis were estimaed in this research. The intratracheal instillation of bleomycin (2 mg/kg, b.w.) significantly altered plasma electrolytes, including sodium, magnesium, and potassium contents. However, treatment with various doses of ECAE resulted in a significant improvement (*p* < 0.05) in the electrolytic parameters in a dose dependent manner, with the higher dose of ECAE (100 mg/kg, b.w.) showing a more substantial effect. These results highlight the therapeutic potential of ECAE against BLEO‐induced electrolytic homeostasis disturbances (Table 4).

**TABLE 4:** Effect of bleomycin (BLEO) instillation and *Eucalyptus cinerea* seeds aqueous extract (ECAE)/gallic acid (GA) on bleomycin (BLEO)‐induced changes in plasma sodium, magnesium and potassium contents.

Group	Sodium (mg/dL)	Magesium (mg/L)	Potassium (mg/dL)
H_2_O	345.34 ± 4.49	29.95 ± 0.90	13.95 ± 1.16
BLEO	257.33 ± 7.59*	45.59 ± 3.53*	62.93 ± 2.67*
ECAE‐50	304.16 ± 5.99#	36.28 ± 1.53#	19.62 ± 2.94#
ECAE‐100	327.97 ± 5.45#	32.40 ± 1.08#	17.24 ± 1.88#
GA	325.51 ± 6.58#	31.58 ± 2.43#	15.75 ± 1.91#

*Note*: H_2_O: Normal control group, BLEO: Intratracheal instilled bleomycin group; ECAE: *Eucalyptus cinerea* seeds aqueous extract at different doses (50 and 100 mg/kg) after BLEO instillation. Data are expressed as means ± SD (*n* = 6); *p < 0.05 compared to control group and #*p* < 0.05 compared to BLEO group (ANOVA test).

## 4 DISCUSSION

Food‐derived natural products have been shown to exert inhibitory actions potential on pulmonary fibrosis (PF) by reducing reactive oxygen species (ROS) and blocking inflammatory pathways (Wang, 2023). In the present research study, the nutritional value and chemical composition of seeds from *Eucalyptus cinerea* and the ameliorative actions of ECAE against BLEO‐induced pulmonary fibrosis in rats were evaluated.

The LC‐MS analysis allowed us to identify seven phenolic compounds belonging to three main classes of polyphenols, such as phenolic acids, flavonoids, and tannins. The bioaccessibility of phenolic compounds has been the subject of many recent reviews. In fact, the bioavailability of tannic acids was evaluated in vitro through ligated rat small intestine segments; and as a result, this compound was absorbed by the intestinal wall at a proportion of 50% (Carbonaro et al., 2001). Phenolic acids with small‐molecular weight, such as gallic acid and isoflavones, are easily absorbed through the tract, as well as flavones, catechins, and quercetin glucosides (Martin & Appel, 2010).

Importantly, our investigation exposed that ECAE exhibited strong scavenging capacity against both DPPH^•^ and H_2_O_2_ radicals. These antioxidant capacities could be attributed to high levels of phenolic compounds, such as flavonoids and total/condensed tannins in ECAE. Our results are in line with those of the literature (Elkolli et al., 2023). It has been demonstrated that polyphenolic substances, such as ellagic acid and epicatechin, are particularly plentiful in the *Eucalyptus* genus and are responsible for the antioxidant effects of its extracts (Bhuyan et al., 2015). Additionally, ellagic acid is primarily responsible for this powerful antioxidant activity (Dhakad et al., 2018). Other research has found that flavonoids increase the likelihood of antioxidant activity in *Eucalyptus* extracts (Parham et al., 2020).

Pulmonary fibrosis (PF) induced by intratracheal instillation of bleomycin is one of the most widely used models for lung diseases. BLEO instillation (2 mg/kg body weight) was chosen to induce a mild‐to‐moderate PF, that could be more amenable to nutritional prevention. In these conditions, BLEO significantly reduced food/water intake and body weight gain, on the other hand, it increased relative lung weight. An increase in organ mass, often due to edema, cellular hypertrophy, or immune cell infiltration, is a key indicator of acute or chronic inflammation. This swelling signals an active immune response (Jedidi et al., 2023). Consumption of *Eucalyptus cinerea* seeds aqueous extract by BLEO‐treated rats alleviated these alterations and led to control values at 100 mg/kg. The protective effects of polyphenol‐rich extracts on lungs weighs have been previously observed in PF rats (Bahri et al., 2023; Dhaouafi et al., 2023).

Histological examination of lung samples from BLEO‐intoxicated rats revealed severe alveolar structural damage, a large number of inflammatory cell infiltration and fibroblast hyperplasia, a significantly enlargement of the alveolar septum, and extensive deposition of collagen fibers in the alveolar septum. Furthermore, this damage was accompanied by a significant increase in the fibrosis score, as previously described (Abidi et al., 2017; Ochi et al., 2025). These alterations were significantly and dose‐dependently corrected by *Eucalyptus* seeds aqueous extract treatment. Furthermore, the beneficial effects of various polyphenols and diet on pulmonary histological structure have been widely reported. It has been previousley documented that gallic acid improves BLEO‐induced histopathological lesions, while fish oils decrease lung erosion in a *Pseudomonas aeruginosa* model (Caron et al., 2015; Nikbakht et al., 2015).

CRP and WBCs are commonly used as markers of inflammation. In PF, BLEO stimulates immune cells in the lungs, leading to the increase of inflammatory markers such as CRP and ALP. Furthermore, BLEO stimulates macrophages to produce chemokines, which attract additional inflammatory cells, such as lymphocytes, monocytes, neutrophils, and eosinophils, to the lung tissue, aggravating the inflammatory response.

In this study, we evaluated the influence of ECAE on an experimental rats model of PF caused by BLEO. ECAE consumption successfully reduced lung tissue inflammation and fibrosis, repaired damaged alveolar structure, lowered immune cells, and alleviated lung tissue damage. These findings are in line with previous research studies (Abidi et al., 2022; Bahia et al., 2024).

Moreover, the increased MDA level induced by BLEO (2 mg/kg, b.w.) stimulates lipid peroxidation, leading to the release of cell contents, cross‐linking of proteins and nucleic acid molecules, cell toxicity, and finally death (Guan et al., 2018). In our research, pulmonary and plasmatic MDA content decreased significantly after treatment of rats with ECAE. Studies carried out with herbal extracts such as *Marrubium vulgare* (Abidi et al., 2022), or purified polyphenols such as gallic acid have also reported a decrease of pulmonary lipoperoxidation (Nikbakht et al., 2015).

Our findings demonstrate that pulmonary and plasmatic antioxidants contents (GSH and –SH groups) and SOD, CAT and GPx activities were significantly decreased by BLEO intoxication. However, ECAE/GA administration increased the activity of these endogenous antioxidant enzymes. Because phenolic compounds are known for their antioxidant properties (Vassilina et al., 2025), we evaluated the antioxidative defenses that could play an important role in macromolecule protection against oxidation. A beneficial effect of polyphenols on SOD, CAT and GPx activities has previously been reported in various models. In the idiopathic pulmonary fibrosis (IPF) model, supplementation with *Marrubium vulgare* exerted an increase of SOD, CAT and GPx activities and antioxidants (GSH and –SH) contents (Abidi et al., 2022). Additionally, the potential in vitro antioxidant power of ECAE against DPPH^•^ and H_2_O_2_ confirms the in vivo antioxidant capacity observed in the present study.

The obtained results demonstrated a significant increase in intracellular mediators such as hydrogen peroxide and calcium in plasma and lung tissues in response to BLEO‐induced oxidative stress. PF caused an accumulation of Ca in pulmonary cells, triggering increased ROS production, reducing cell viability, and leading to cell death (Feno et al., 2019). Interestingly, treatment with ECAE and GA effectively restored the Ca imbalance by activating the calcium pumping system, a mechanism observed with other plant extracts rich in phenolic compounds, such as *Olea europaea* (Bahri et al., 2023). More importantly, the mechanism of action of ECAE can be explained by its bronchodilator effect following the decrease in Ca^2+^ level, hence the dilation of the bronchial muscles. In the same respect, certain polyphenols, such as quercetin, have been shown to have bronchodilator effects, relaxing the muscles of the airways and facilitating breathing (Emran et al., 2024). It has been demonstrated that phenolic compounds act by blocking the CaSR receptor, which prevents the polyamine‐induced increase in intracellular Ca^2+^, thereby reducing the proliferation of pulmonary fibroblasts (Wolffs et al., 2025).

It has also been suggested that treatment with ECAE protects against the overload of pulmonary cells with H_2_O_2_ caused by the single administration of BLEO. However, this element combined with free iron is involved in the generation of the hydroxyl radical (OH^•^) via the Fenton reaction, which plays a major role in oxidative damage by affecting molecular structures. In this respect, it has been documented that living organisms create a complex endogenous and exogenous antioxidant defense system to protect against ROS (Pisoschi & Pop, 2015).

Finally, we observed that BLEO instillation caused an imbalance in plasma electrolyte levels. This toxic agent increased magnesium and potassium levels, and in turn, a reduction in sodium levels. Indeed, in previous reports, researchers observed that inflammation was associated with a tendency toward hypochloremia, hypokalemia, and metabolic alkalosis, with more pronounced hyponatremia (Scurati‐Manzoni et al., 2014). However, treatment with increased concentrations of ECAE significantly and dose‐dependently corrected these disorders. This regulation is due to the richness of *Eucalyptus* seeds in polyphenols. Additionally, phenolic acid and tannic acid were able to inhibit Na^+^‐K^+^‐ATPase, responsible for maintaining the sodium and potassium gradients, as supported by literature findings (Babu et al., 2006; Heger et al., 2022).

## 5 LIMITATIONS

This research investigates the beneficial effect of functional foods from *Eucalyptus cinerea* seeds on pulmonary fibrosis (PF), conducted on an animal model and validated by the underlying mechanisms. This study has some limitations: (i) Although this study demonstrates that ECAE restores biological homeostasis and exerts anti‐inflammatory effects, its potential role in ameliorating fibrosis remains unverified. Further investigations into antifibrotic biomarkers are therefore warranted to build upon these results. (ii) The anatomical, physiological, bioavailability, and compositional differences of the pulmonary microbiota between rodents and humans constitute limiting factors in the direct application of our results to humans. (iii) Furthermore, the bleomycin‐induced PF model, while frequently used, does not fully reproduce the complexity of human fibrosis, which can have diverse etiologies such as infections, dust exposure, inhalation of molds, bacteria, or viruses, certain autoimmune diseases, and side effects of medications or treatments such as radiotherapy. Therefore, while our findings offer valuable insights into the therapeutic potential of *Eucalyptus cinerea*, further clinical trials are essential to validate them and assess human applicability. (iv) Finally, future studies should evaluate how ECAE affects adrenergic receptors, as it seems to antagonize cholinergic bronchoconstriction or mimic beta‐2‐adrenergic effects, leading to sympathetic‐like bronchodilation.

## 6 CONCLUSION

In the present study, we characterized the phytochemical profile of *Eucalyptus cinerea* seeds and provided the first evidence of its potential therapeutic effects against pulmonary fibrosis (PF) in a bleomycin (BLEO)‐induced murine model. ECAE demonstrated high levels of polyphenols, flavonoids, and tannins, correlating with significant free radical scavenging activity. It has been demonstrated that ECAE treatment significantly alleviated PF symptoms, notably reducing relative lung weight, oxidative stress, and intracellular mediators in both plasma and lung tissue. These results strongly suggest that *Eucalyptus cinerea* has therapeutic potential for PF, warranting further investigation into its active compounds for developing novel anti‐fibrotic agents and functional food ingredients. However, further clinical investigations are essential to confirm these results and explore their applicability to the treatment of human lung diseases.

## AUTHOR CONTRIBUTIONS

**Saber Jedidi:** Conceptualization; formal analysis; investigation; methodology. **Nourhène Dhawefi:** Conceptualization; formal analysis; methodology. **Anouar Abidi:** Conceptualization; formal analysis; investigation; methodology. **Houcem Sammari:** Conceptualization; data curation. **Ala Ayari:** Conceptualization; formal analysis; methodology; software. **Hichem Sebai:** Conceptualization; data curation; formal analysis; funding acquisition.

## CONFLICT OF INTEREST STATEMENT

The authors declare that they have no known competing financial interests or personal relationships that could have appeared to influence the work reported in this paper.

## ETHICS STATEMENT

Experimental protocols were used in accordance with Tunis University's local ethics committee (University of Jendouba: UJ2021‐03‐4022) in accordance with the International Council of Laboratory Animal Science for the use and care of animals.

## Data Availability

The data that support the findings of the present study are available on request from the corresponding author. The data are not publicly available due to privacy or ethical restrictions.
